# Supplementary material for: Deep learning at the edge enables real-time streaming ptychographic imaging
Source: Nat Commun. 2023 Nov 3;14:7059. doi: 10.1038/s41467-023-41496-z (PMC10624836; doi:10.1038/s41467-023-41496-z)
Supplement: Supplementary file 1 — Supplementary Information [file 41467_2023_41496_MOESM1_ESM.pdf]

# Supplementary Information : Deep learning at the edge enables real-time streaming ptychographic imaging

Anakha V Babu,[1,†] Tao Zhou,[1,†] Saugat Kandel,[1] Tekin Bicer,[1] Zhengchun Liu,[1] William Judge,[2] Daniel J. Ching,[1] Yi Jiang,[1] Sinisa Veseli,[1] Steven Henke,[1] Ryan Chard,[1] Yudong Yao,[1] Ekaterina Sirazitdinova,[3] Geetika Gupta,[3] Martin V. Holt,[1] Ian T. Foster,[1] Antonino Miceli,[1,*] Mathew J. Cherukara [1,*]

[1]Argonne National Laboratory, 9700 S Cass Ave, Lemont, IL, USA

[2]Formerly at Department of Chemistry, University of Illinois, Chicago, IL, USA

[3]NVIDIA Corporation, Santa Clara, CA, USA

[*]To whom correspondence should be addressed: mcherukara@anl.gov, amiceli@anl.gov

[†]Equal contributions

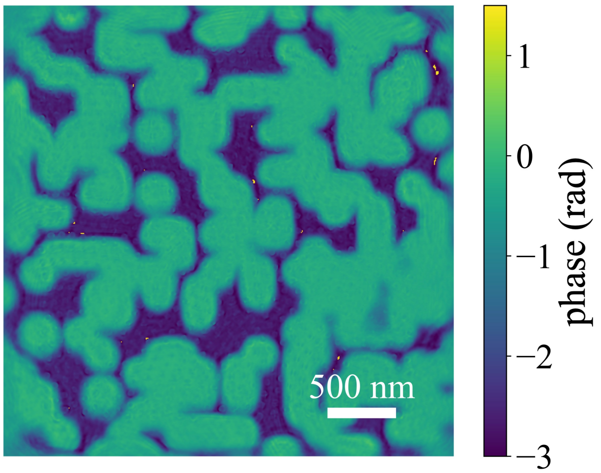


Supplementary Figure 1: **Phase of a typical training data**. The phase was retrieved using iterative methods.

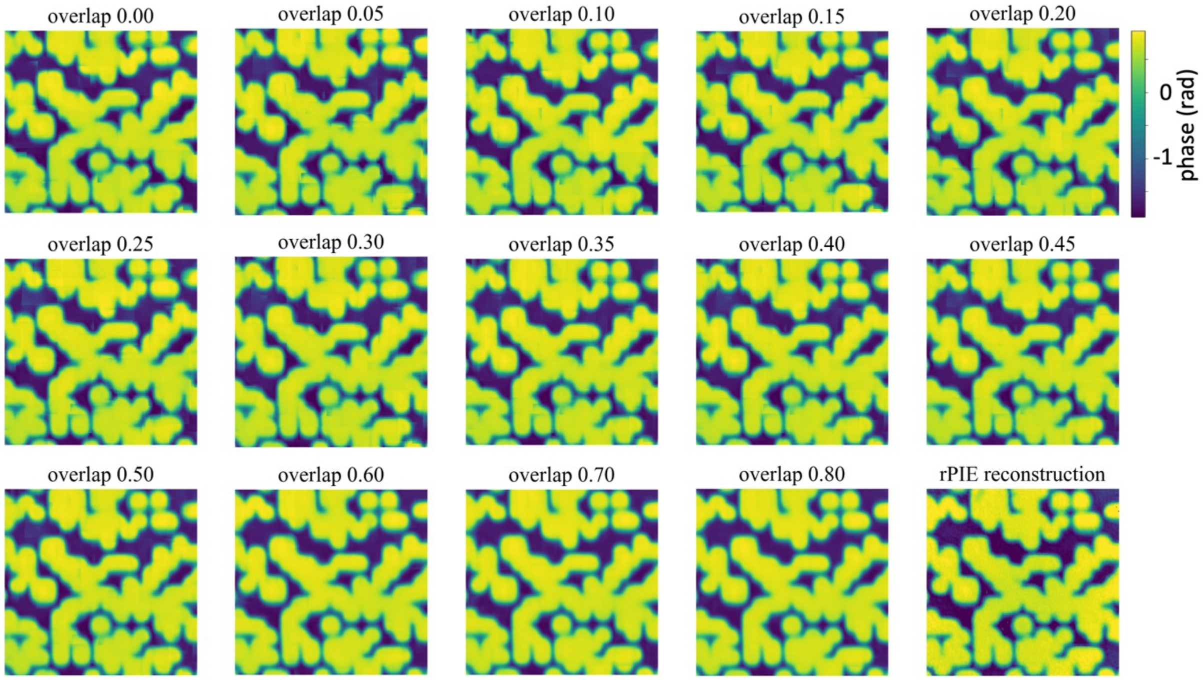


Supplementary Figure 2: **Stitched NN inference at various reduced overlap**. Results of iterative phase retrieval is also shown for comparison. To create scans with overlap ratio between 0 and 0.5, we selectively remove data points from a parent scan with an initial overlap of 0.6 until the average overlap matches the desired value. To create scans with overlap ratio between 0.6 and 0.8, we selectively remove data points from another parent scan with an initial overlap of 0.9 until the average overlap matches the desired value.

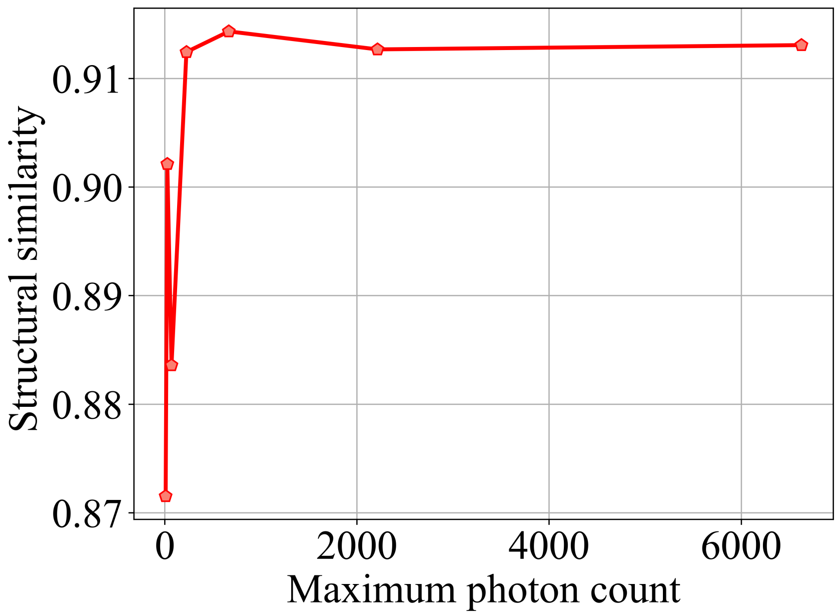


Supplementary Figure 3: **Accuracy of the NN inference with down scaling of the intensity.** Performance of the workflow when the diffraction intensities are logarithmically scaled down with scaling factors starting from 10 to 10,000. The x-axis represents the maximum photon count per diffraction pattern at each of the scaling factors. It can be observed that the AI inference can retain a structural similarity above 80% when the maximum photon count per diffraction pattern is as low as 10. Source data are provided as a Source data file.

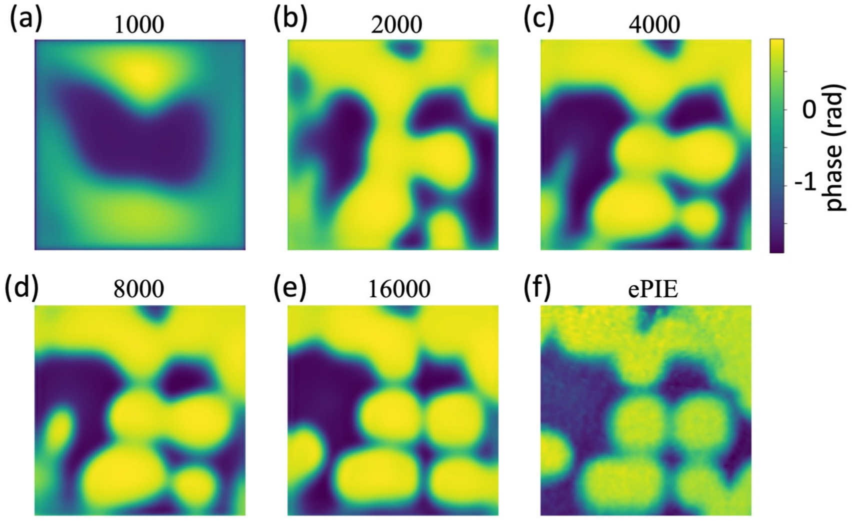


Supplementary Figure 4: **Effect of continual learning.** Evolution of the AI-inferred phase after training on (a) 1000, (b) 2000, (c) 4000, (d) 8000 and (e) 16000 sets of data. The result from iterative phase retrieval is shown in (f) serving as the ground truth.

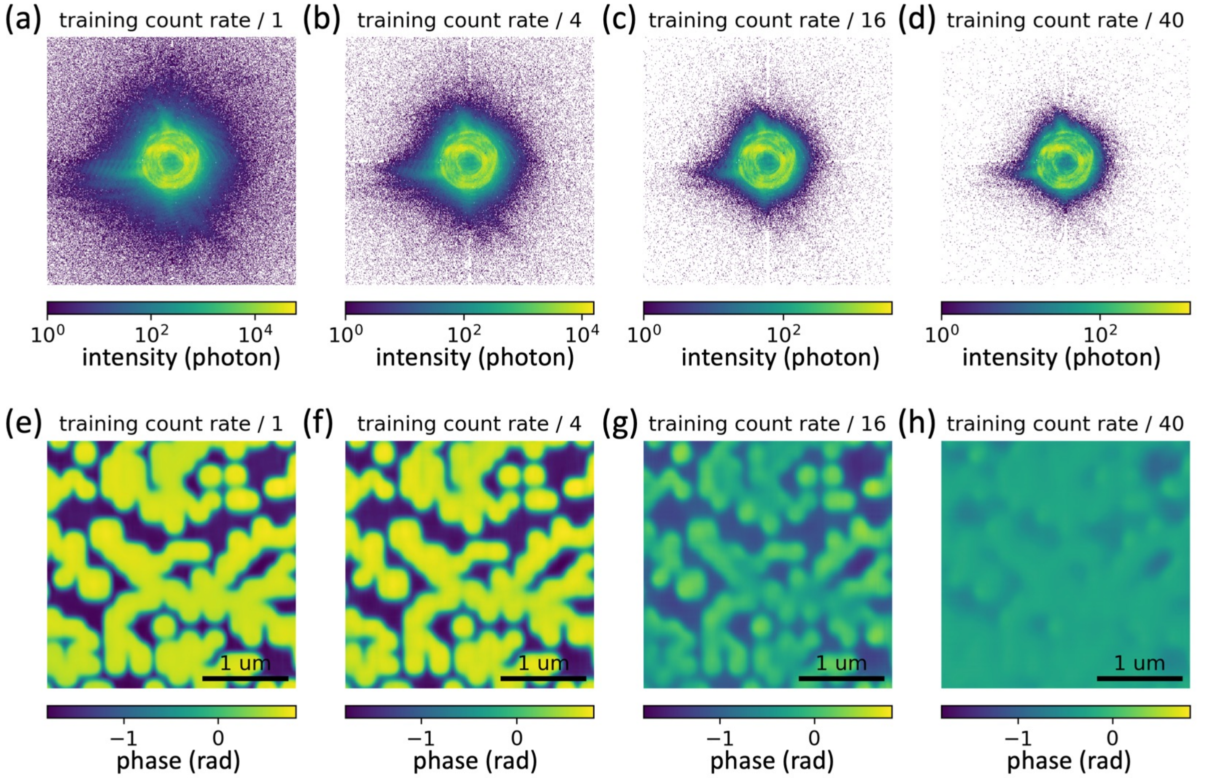


Supplementary Figure 5: **Tolerance of the trained model to changes in count rate.** (a-d) example experimental diffraction patterns acquired on the same area of the sample, at different count rates. (e-h) stitched phase inference from the same model on data acquired at different count rates. The count rate for the trained model is the same as the data shown in (a).

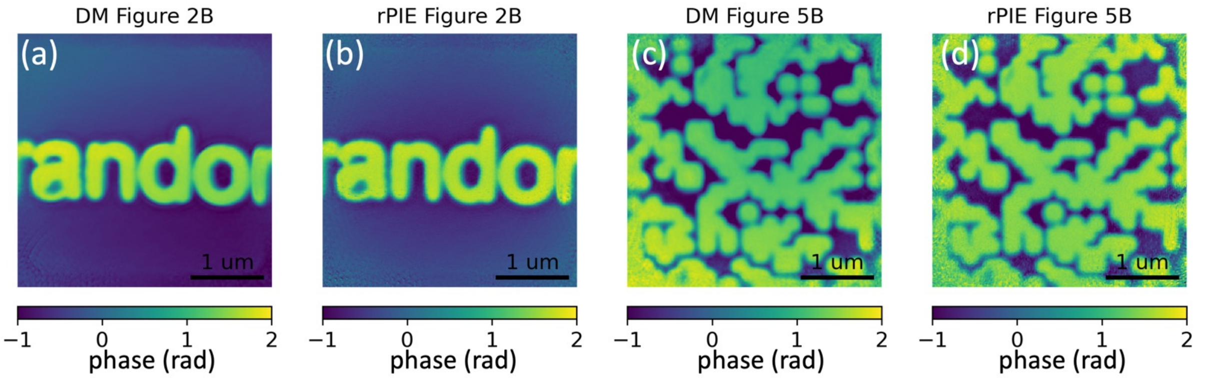


Supplementary Figure 6: **Comparison between DM and rPIE.** (a) DM and (b) rPIE retrieved phase for data shown in Figure 2B. (c) DM and (d) rPIE retrieved phase for data shown in Figure 5B.

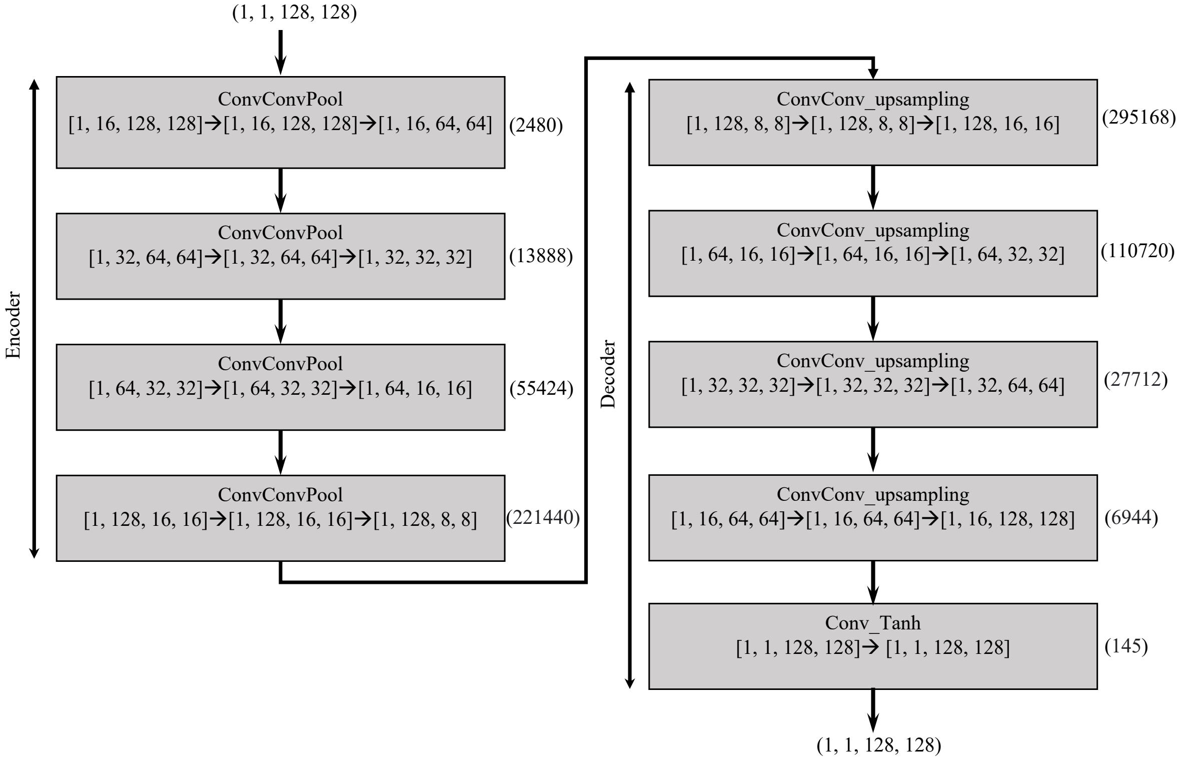


Supplementary Figure 7: **Architecture of PtychoNN 2.0.** Detailed architecture of PtychoNN 2.0 with a fully convolutional autoencoder network indicating different convolutional layers and their corresponding output shapes. The convolution layers use a filter size of 3x3 and a stride of 1. Here 'Conv' refers to 2D convolution followed by a ReLU activation function, 'Pool' corresponds to a 2D maxpooling operation with a pool size of $2\times2$, and 'upsampling' uses a 2D bilinear interpolation for a $2\times2$ input. The final 'TanH' activation layer outputs predictions in the range [-1,1], which are then scaled to get output phases in the range [-$\pi$, $\pi$]. The output shapes in each block are read as [batch size, number of filters, height, width]. The number of trainable parameters is also indicated in parenthesis against each block in the figure.

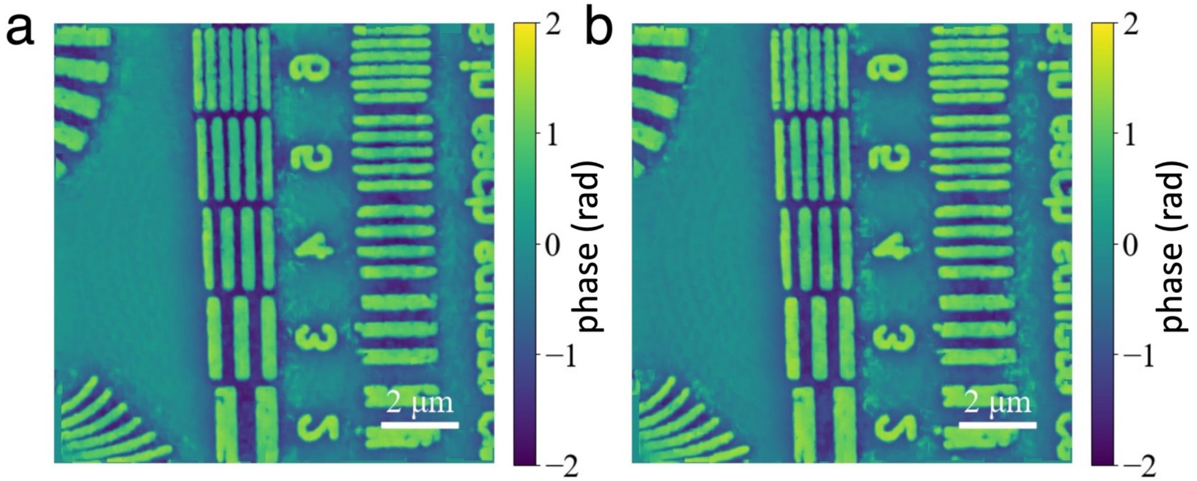


Supplementary Figure 8: **Comparison between the orignal PtychoNN and PtychoNN 2.0.** Qualitative comparison of inferences by two different models of PtychoNN, **a** having 4.8 M trainable parameters, and **b** PtychoNN 2.0 with 0.7 M trainable parameters.

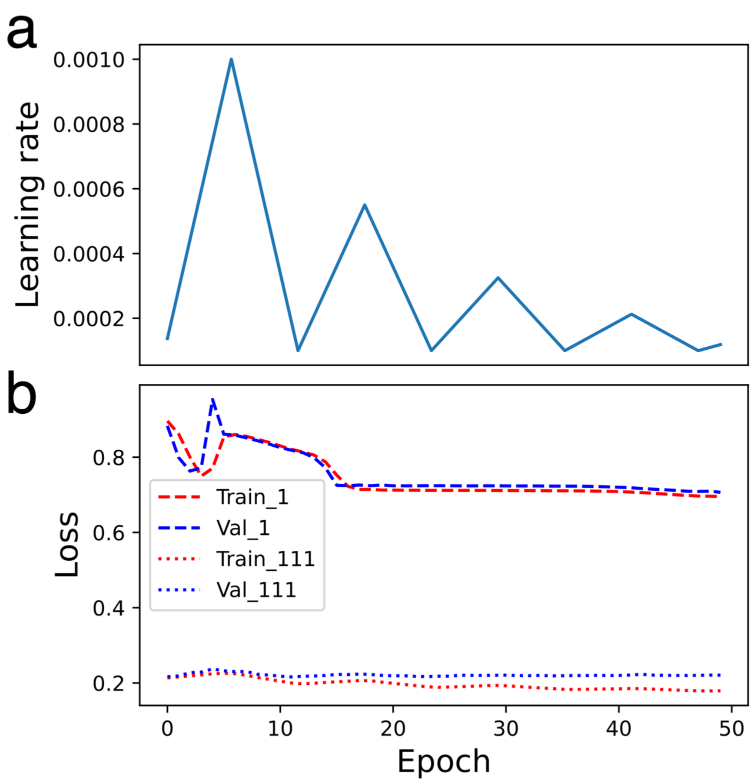


Supplementary Figure 9: **Evolution of training and validation losses.** **A** Cyclic learning rate policy adopted for the training with an initial value of 0.0001. The maximum and minimum learning rates are 0.001, 0.0001 respectively. **B** Variation in training and validation loss over 50 epochs estimated at two different iterations of the continual learning approach when ptychography scan #1 and scan #111 were added to the existing training data. Source data are provided as a Source data file.

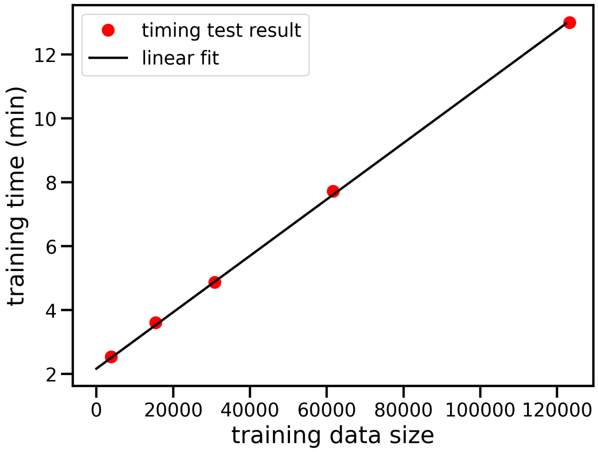


Supplementary Figure 10: **Training time versus training data size on 8 A100 GPUs.** Source data are provided as a Source data file.

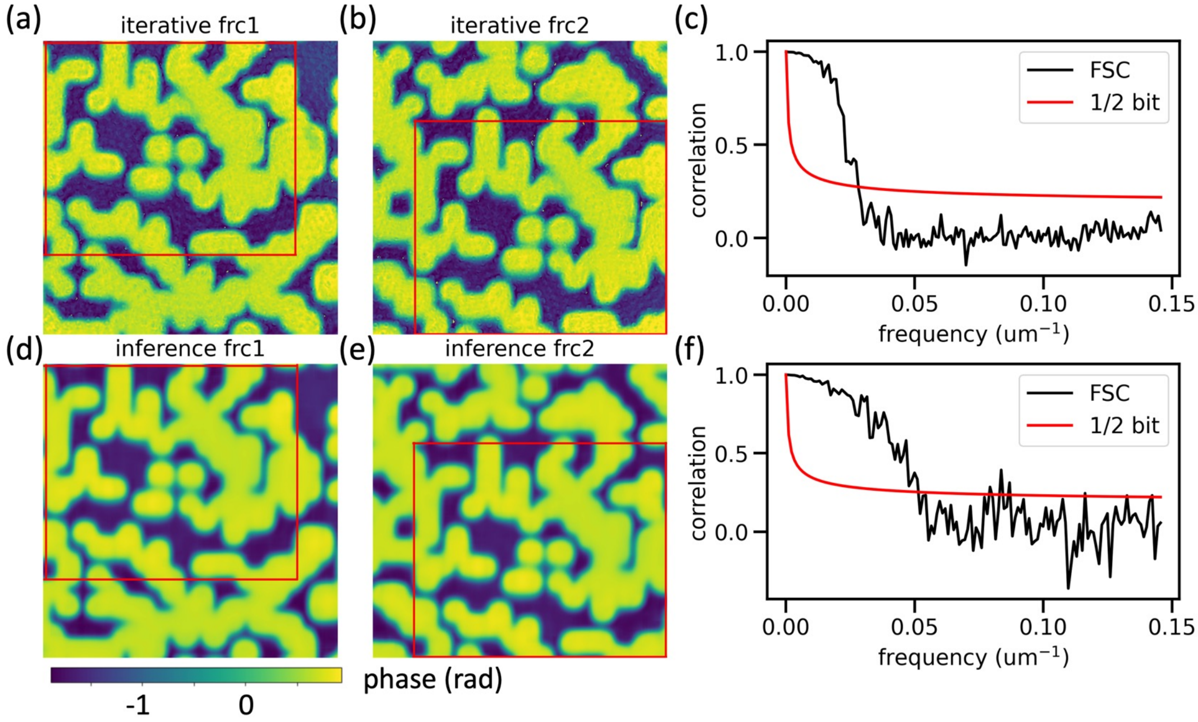


Supplementary Figure 11: **Fourier Ring Correlation on the iteratively retrieved and stitched NN inference.** Iteratively retrieved phase for (a) scan frc1 and (b) scan frc2. The red rectangle marks the common area (c) Fourier shell correlation calculation was performed. Stitched inference for (d) scan frc1 and (e) scan frc2. The red rectangle marks the common area (f) Fourier shell correlation calculation was performed. Source data are provided as a Source data file.
